# Osimertinib-induced Keratitis and Secondary Toxic Epidermal Necrotic Drug Eruption- A Case Report and Literature Review

**DOI:** 10.2174/1574886318666230529123200

**Published:** 2023-11-10

**Authors:** Yunhua Xu, Yong Li, Jie Luo, Rong Tang

**Affiliations:** 1 Department of Oncology, Yi Ling Hospital of Yichang, Hubei Province 443002,China;; 2 Department of Dermatology, The Second People's Hospital of Yichang, Hubei Province 443001,China

**Keywords:** Osimertinib, EGFR, SJS/TEN, keratitis, target-therapy, oncology

## Abstract

**Background:**

Osimertinib is a third-generation Tyrosine Kinase inhibitor, mainly used in non-small cell lung cancer with EGFR mutation. Its efficacy and safety have been confirmed by clinical practice. Toxic epidermolysis necrotizing disease (TEN) is a severe drug eruption that is rare in clinics and has a high mortality rate. Toxic epidermal necrotic drug rash caused by Osimeritinib is even rarer.

**Objective:**

To investigate the rare side effects of Osimertinib through a case of toxic Epidermal necrosis

**Case Presentation:**

A 63-year-old female patient was diagnosed with lung adenocarcinoma with brain metastases, and genetic testing revealed an EGFR21 exon mutation. The disease progressed 24 days after the administration of gefitinib, then the patient switched to Osimertinib (80 mg QD) and, resulting in keratitis and secondary systemic toxic epidermolysis necrotizing disease (TEN). Finally, the patient died.

**Conclusion:**

Although the clinical use of osimertinib is becoming widespread, the side effects may not be fully understood. Clinicians should pay more attention to the occurrence of the side reaction and deal with it in time.

## 1 INTRODUCTION

A 63-year-old female patient visited our outpatient clinic for dizziness and fatigue on November 04. Chest-enhanced CT showed a nodule (1.3×1.9 cm) in the anterior segment of the right upper lobe of the lung. Considering neoplasm and needle biopsy is recommended. Head MRI showed multiple abnormal enhancement signals in the right occipital lobe and bilateral parietal lobe, which suggested the possibility of metastasis.

## 2 CASE PRESENTATION

**Fig. (1):**
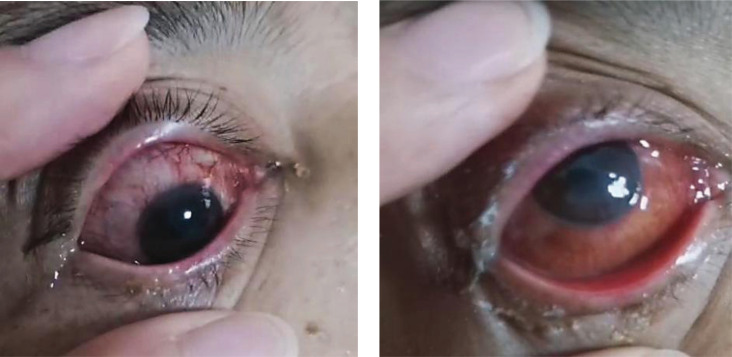
Scleral congestion and corneal ulcer.

**Fig. (2):**
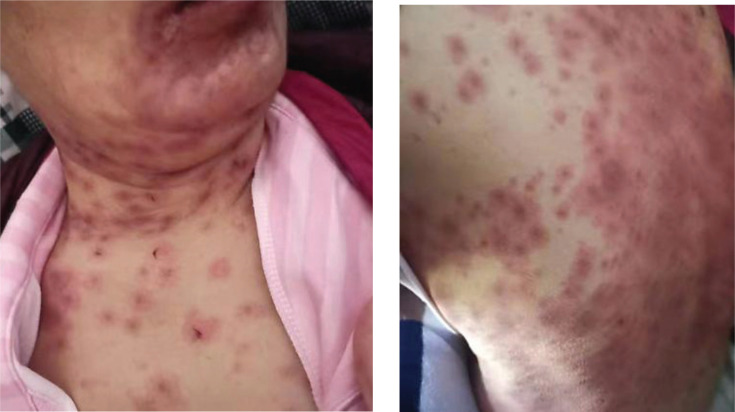
Skin erythema and rash.

**Fig. (3):**
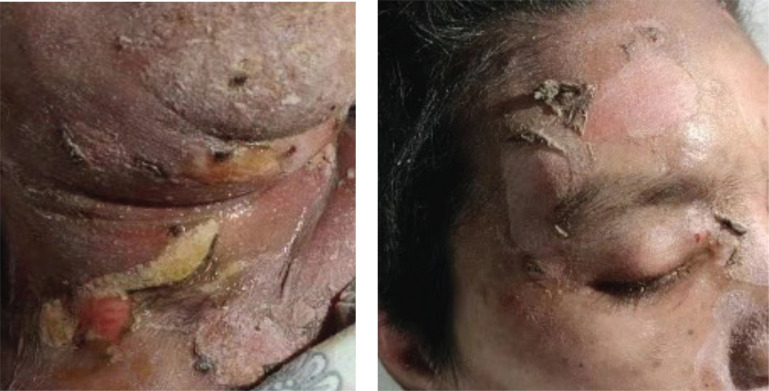
Skin peeling off the whole body.

Thoracoscopic wedge resection of the right upper lobe was performed on November 17, 2020. Pathological examination showed adenocarcinoma in the right upper lung, which was mainly adherent type, and a few were acinar type. The EGFR21 exon L858R mutation was detected. On November 23, 2020, oral administration of Gefitinib (250mg QD) was started, and no obvious diarrhea or rash occurred during the treatment. On December 17, 2020, the patient was admitted to our hospital again due to headache, nausea, and vomiting. Emergency head CT showed multiple low-density lesions in the brain. Considering the progression of intracranial lesions and the patient refused radiotherapy, she was switched to Osimertinib (80 mg QD). One week later, the patient felt that the symptoms of headache, nausea, and vomiting were significantly relieved. On December 29, 2020, the patient complained of blurred vision, red vision, and photophobia in the right eye. After an ophthalmological consultation, the patient was diagnosed with keratitis (Fig. **1**), which was considered to be related to Osimertinib. Osimertinib was discontinued and according to the consultation, she was treated with basic fibroblast growth factor eye drops and tobramycin eye drops. On January 02, 2021, the patient began to develop rashes (neck, chest, abdominal wall, lower back, bilateral upper limbs, bilateral thighs) (Fig. **2**) with obvious itching, a local large area of blisters, and then the skin is peeled off (Fig. **3**).

The treatment was ineffective, including local dressing change, immunoglobulin infusion, and anti-infection. The patient died on January 11, 2021.

## 3 DISCUSSION

Osimertinib is a third-generation tyrosine kinase inhibitor. Since it was first approved by US FDA in 2015 as a targeted therapy for locally advanced or metastatic non-small cell lung cancer with EGFR T790M mutation, its clinical research and application have been widely carried out. AURA3 study showed that for non-small cell lung cancer with T790M mutation, the overall response rate of Osimertinib reached 71%, and the median PFS was 10.1 months [1]. Based on the results of the FLAURA study published in October 2017, the National Comprehensive Cancer Network guidelines of the United States recommended Osimertinib as the first choice for the treatment of advanced non-small cell lung cancer with EGFR mutations in 2019 [2]. The meta-analysis by Jing Liu *et al.* showed that the top three side effects of Osimertinib were: rash, with an incidence of 42%; diarrhea, an incidence was 35%, paronychia was 30%. The side effect of more than grade three was pneumonia, with an incidence of 3%. The incidence of anorexia was 2%, and rash and diarrhea were 1%. Osimertinib is generally considered to be safe and well-tolerated [3].

Stevens-johnson Syndrome (SJS) and toxic epi-necrolysis (TEN) are severe mucocutaneous diseases. The typical clinical manifestations are hemorrhagic erythema of the skin/mucosa, blisters on the erythema, and severe epidermal necrolysis. According to the suffered skin area of the patient, the exfoliation area of less than 10% of the body surface area was SJS, more than 30% was TEN, and between 10% and 30% was SJS/TEN overlap.SJS and TEN are a group of delayed hypersensitivity reactions, and the incidence of TEN is generally believed to be 1.5-2.5 per million [4]. Drugs are the most important (95%) pathogenic factors [5], including non-steroidal anti-inflammatory and analgesic drugs, antiepileptic drugs, allopurinol, *etc*. [6].

The pathogenesis may include:

a) Apoptosis mechanism: drugs can be directly integrated into major histocompatibility complex-l (MHC-1) and T cell receptors, leading to the clonal proliferation of specific cytotoxic T cells. Proliferating cytotoxic T cells release soluble apoptotic mediators, which further apoptotic keratinocytes [7].

b) Keratinocyte apoptotic mediators: In the process of keratinocyte apoptosis, it may not only be the killing effect of drug-specific cytotoxic T cells and NK cells but also a large number of apoptotic mediators released by them in this process, which changes the anti-apoptotic pathway of the body and causes the body to change or defect in the monitoring of the drug-specific immune response. A variety of cytotoxic proteins and cytokines have been confirmed to be involved in the process of keratinocyte apoptosis in TEN patients, including perforin/granzyme B, Fas/FasL apoptosis-related factor ligand, tumor necrosis factor-α, and granolysin [8].

c) Genetic factors: Recent studies have shown that the allele HLA-B*1502 is closely associated with carbamazepine-induced SJS and TEN in Chinese, especially the Han population [9], and also with other antiepileptic drugs induced SJS and TEN [10]. The HLA-B*5801 allele is closely related to allopurinol-induced SJS and TEN [11]. Treatment methods include early withdrawal of suspected allergenic drugs, strengthening of supportive therapy, intravenous immunoglobulin infusion, *etc*. [12]. Literature reports that the mortality rate of TEN is 25%-35% [13], and the prognosis is grim.

TEN caused by Osimertinib is rare. After a literature search, only 2 cases were reported in the world up to April 2022. One case was reported by Chinese Wang *et al.* [14], who recovered after active treatment. One case was reported by Sato [15] in Japan, and the patient also recovered. This patient with EGFR21 L858R mutation had no side effects after 4 weeks of oral Gefitinib. Due to the progression of intracranial lesions, oral Osimertinib 80 mg daily was given for 7 days, and the cranial nerve symptoms were relieved. However, corneal ulcers and oral mucositis developed after the drug was stopped, the skin lesions worsened, and the skin exfoliation was extensive throughout the body. According to the ALDEN (algorithm of drug causality for epidermal necrolysis) scoring standard [16], the score was 4 points, which was considered to be highly related to Osimertinib.

## CONCLUSION

Although the clinical application of Osimertinib is gradually expanding, the understanding of its side effects may not be enough, and the three cases reported so far, including the present case, are of Asian race. Whether genetic factors, especially HLA-B-related genes, also play a key role in the process of Osimertinib causing TEN deserves further study. The incidence of SJS/TEN caused by osimertinib is very low, but due to the high mortality and harmfulness of SJS/ TEN, clinicians should carefully observe skin reactions during the use of osimertinib in patients and deal with it in time.

## Data Availability

All the data and supportive information are provided within the article.

## LIST OF ABBREVIATION

SJS: Stevens-Johnson Syndrome
MHC-1: Major Histocompatibility Complex-l
ALDEN: Algorithm of Drug Causality for Epidermal Necrolysis

## References

[1] Mok T.S., Wu Y.L., Ahn M.J. (2017). Osimertinib or Platinum-Pemetrexed in EGFR T790M-positive lung cancer.. N. Engl. J. Med..

[2] Soria J.C., Ohe Y., Vansteenkiste J. (2018). Osimertinib in untreated EGFR-mutated advanced non-small-cell lung cancer.. N. Engl. J. Med..

[3] Liu J., Li X., Shao Y., Guo X., He J. (2020). The efficacy and safety of osimertinib in treating nonsmall cell lung cancer.. Medicine.

[4] Hsu D.Y., Brieva J., Silverberg N.B., Silverberg J.I. (2016). Morbidity and mortality of Stevens-Johnson syndrome and toxic: Epidermal necrolysis in United States adults.. J. Invest. Dermatol..

[5] Lerch M., Mainetti C., Beretta-Piccoli B.T., Harr T. (2018). Current perspectives on Stevens-Johnson syndrome and toxic epidermal necolysis.. Clin. Rev. Allergy Immunol..

[6] Lerch M., Mainetti C., Terziroli Beretta-Piccoli B., Harr T. (2018). Current perspectives on Stevens-Johnson syndrome and toxic epidermal necrolysis.. Clin. Rev. Allergy Immunol..

[7] Nassif A., Bensussan A., Boumsell L. (2004). Toxic epidermal necrolysis: Effector cells are drug-specific cyto-toxic T cells.. J. Allergy Clin. Immunol..

[8] Wolkenstein P., Wilson Y.T. (2016). Toxic epidermal necrolysis: The past, the guidelines and challenges for the future.. J. Plast. Reconstr. Aesthet. Surg..

[9] Chung W-H., Hung S-I., Hong H-S. (2004). A marker for Stevens–Johnson syndrome.. Nature.

[10] Hung S.I., Chung W.H., Liu Z. (2010). Common risk allele in aromatic antiepileptic-drug induced Stevens-Johnson syndrome and toxic epidermal necrolysis in Han Chinese.. Phamacogenomics.

[11] Hung S.I., Chung W.H., Liou L.B. (2005). HLA-B*580l allele as a genetic marker for severe cutaneous adverse reactions caused by allopurinol.. Proc. Natl. Acad. Sci..

[12] Enk A.H., Hadaschik E.N., Eming R. (2016). European Guidelines (S1) on the use of high-dose intravenous immunoglobulin in dermatology.. J. Eur. Acad. Dermatol. Venereol..

[13] Harr T., French L.E. (2010). Toxic epidermal necrolysis and Stevens-Johnson syndrome.. Orphanet J. Rare Dis..

[14] Wang J., Cheng X., Lu Y., Zhou B. (2018). A case report of toxic epidermal necrolysis associated with AZD-9291.. Drug Des. Devel. Ther..

[15] Sato I., Mizuno H., Kataoka N. (2020). Osimertinib-associated toxic epidermal necrolysis in a lung cancer patient harboring an EGFR Mutation-A case report and a review of the literature.. Medicina.

[16] Sassolas B., Haddad C., Mockenhaupt M. (2010). ALDEN, an algorithm for assessment of drug causality in Stevens-Johnson Syndrome and toxic epidermal necrolysis: comparison with case-control analysis.. Clin. Pharmacol. Ther..

